# Effects of Maternal Exposure to Di-(2-ethylhexyl) Phthalate during Fetal and/or Neonatal Periods on Atopic Dermatitis in Male Offspring

**DOI:** 10.1289/ehp.11191

**Published:** 2008-04-09

**Authors:** Rie Yanagisawa, Hirohisa Takano, Ken-ichiro Inoue, Eiko Koike, Kaori Sadakane, Takamichi Ichinose

**Affiliations:** 1 Environmental Health Sciences Division, National Institute for Environmental Studies, Tsukuba, Japan; 2 Department of Health Sciences, Oita University of Nursing and Health Sciences, Oita, Japan

**Keywords:** atopic dermatitis, di-(2-ethylhexyl) phthalate, eosinophils, eotaxin, mast cells

## Abstract

**Background:**

Di-(2-ethylhexyl) phthalate (DEHP) has been widely used in polyvinyl chloride products and is ubiquitous in developed countries. Although maternal exposure to DEHP during fetal and/or neonatal periods reportedly affects reproductive and developmental systems, its effects on allergic diseases in offspring remain to be determined.

**Objectives:**

In the present study, we examined whether maternal exposure to DEHP during fetal and/or neonatal periods in NC/Nga mice affects atopic dermatitis-like skin lesions related to mite allergen in offspring.

**Methods:**

We administered DEHP at a dose of 0, 0.8, 4, 20, or 100 μg/animal/week by intraperitoneal injection into dams during pregnancy (gestation days 0, 7, and 14) and/or lactation (postnatal days 1, 8, and 15). Eight-week-old male offspring of these treated females were injected intradermally with mite allergen into their right ears. We then evaluated clinical scores, ear thickening, histologic findings, and protein expression of eotaxin in the ear.

**Results:**

Maternal exposure to a 100-μg dose of DEHP during neonatal periods, but not during fetal periods, enhanced atopic dermatitis-like skin lesions related to mite allergen in males. The results were concomitant with the enhancement of eosinophilic inflammation, mast cell degranulation, and protein expression of eotaxin in overall trend.

**Conclusion:**

Maternal exposure to DEHP during neonatal periods can accelerate atopic dermatitis-like skin lesions related to mite allergen in male offspring, possibly via T helper 2 (T_H_2)-dominant responses, which can be responsible, at least in part, for the recent increase in atopic dermatitis.

Epidemiologic studies have shown that the prevalence of allergic diseases has increased at a great rate, mainly among children and juveniles, over the past several decades (Beasley et al. 2003). The etiology of allergy includes genetic factors (e.g., sensitivity of hosts) and environmental factors (e.g., allergen load, environmental pollutants) (Burney et al. 1990; Peat and Li 1999). In particular, environmental chemicals may increase the potency of allergens and thereby play a role in the development and/or enhancement of allergic diseases (Casillas et al. 1999). In fact, we and other groups have previously reported that diesel exhaust particles, which are major environmental pollutants in urban areas and contain a variety of organic chemicals such as polyaromatic hydrocarbons, possess adjuvant activity (Diaz-Sanchez 1997; Diaz-Sanchez et al. 1994; Heo et al. 2001) and aggravate allergic airway inflammation in murine models (Ichinose et al. 2004; Inoue et al. 2007; Miyabara et al. 1998; Sadakane et al. 2002; Takano et al. 1997, 1998; Yanagisawa et al. 2006).

Di-(2-ethylhexyl) phthalate (DEHP), another environmental chemical, has become ubiquitous in developed countries. DEHP is the most abundant phthalate plasticizer in polyvinyl chloride (PVC) formulations, including vinyl flooring, wall covering, food containers, gloves, and infant toys. However, DEHP is not chemically bound to PVC and thus leaches out from the PVC items with time and use. An epidemiologic study has shown that DEHP in house dust is associated with allergic asthma in children (Bornehag et al. 2004). In animal experiments, DEHP has displayed an adjuvant effect on allergen-related immunoglobulin production (Thor Larsen et al. 2001) and enhanced allergic responses, including the production of interleukin-4 (IL-4) from CD4^+^ T cells (Lee et al. 2004). More recently, we have shown that exposure to DEHP aggravates atopic dermatitis-like skin lesions related to mite allergen in young male mice, as evidenced by macroscopic and microscopic examinations. Furthermore, these changes were consistent with the protein expression of chemokines in the ear tissue in overall trend (Takano et al. 2006).

In contrast, fetuses and infants, who are believed to be one of the most sensitive populations to environmental chemicals (Colborn et al. 1993; Holladay 1999; Holladay and Smialowicz 2000), can be maternally exposed to DEHP. In fact, DEHP and/or mono-(2-ethylhexyl) phthalate (MEHP), a metabolite of DEHP, have been detectable in human cord blood and maternal plasma (Latini et al. 2003a, 2003b). In addition, MEHP has been found in human breast milk (Calafat et al. 2004; Main et al. 2006) and in infant formula (Mortensen et al. 2005; Sorensen 2006). Previous animal studies have suggested that maternal exposure to phthalates during fetal and/or neonatal periods may cause reproductive and developmental toxicities in offspring due to their actions as endocrine-disrupting chemicals (EDCs) (Borch et al. 2006; Kim et al. 2004).

In the present study, we examined whether maternal exposure to DEHP in NC/NgaTndCrj (NC/Nga) mice during fetal and/or neonatal periods affects atopic dermatitis-like skin lesions related to mite allergen in offspring.

## Materials and Methods

### Animals

We purchased 8-week-old male and female NC/Nga mice from Charles River Japan (Osaka, Japan). They were fed a commercial diet (CE-2; Japan Clea Co., Tokyo, Japan) and water *ad libitum* and housed in an animal facility maintained at 22–26°C with 40–69% humidity and a 12/12-hr light/dark cycle. All mice were treated humanely with regard for alleviation of suffering in accordance with guidelines of the National Institute for Environmental Studies for animal experiments. All protocols involving mice were approved by the institutional review board.

### Study protocols

#### Experiment 1: maternal exposure to DEHP during fetal periods

**Figure 1:**
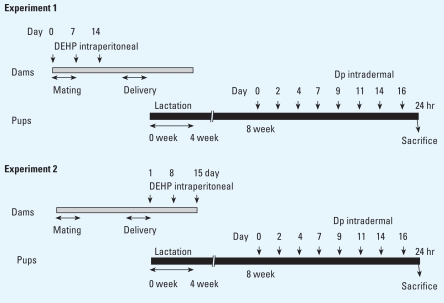
Experimental protocol for maternal exposure to DEHP during fetal and/or neonatal periods and for allergen sensitization in offspring. Dp, *Dermatophagoides pteronyssinus* extract.

We divided male and female mice (8 weeks of age) into seven experimental groups and kept them under specific-pathogen-free (SPF) conditions in an animal facility for 1 week. We mated four male and five female mice in each cage under SPF conditions for 1 week (days 0–7) and then separated male and female mice. We administered DEHP by intraperitoneal injection at a dose of 0.8, 4, 20, or 100 μg dissolved in 0.1 mL olive oil (Wako Pure Chemical Industries, Ltd., Osaka, Japan) to the dams in four groups on days 0, 7, and 14 (Figure 1). These doses were equivalent to daily DEHP intake of 4.8, 24, 120, and 600 μg/kg body weight per day. Two groups of animals received only olive oil and served as vehicle controls, and one group received no treatment (untreated). We checked breeding cages daily for births.

#### Experiment 2: maternal exposure to DEHP during neonatal periods

We divided male and female mice (8 weeks of age) into seven experimental groups and kept them under SPF conditions in an animal facility for 1 week. We mated four male and five female mice in each cage under SPF conditions for 1 week and then separated male and female mice. We checked breeding cages daily for births. We then administered DEHP at a dose of 0.8, 4, 20, or 100 μg by intraperitoneal injection to dams in four groups on days 1, 8, and 15 after birth (Figure 1). Two groups of animals received only olive oil and served as vehicle controls. The untreated group received no treatment.

#### Mite allergen treatment in pups

In both experiments, at least one litter was included in each experimental group. We kept litters with dams until weaning at 4 weeks of age. While under anesthesia with 4% halothane (Takeda Chemical Industries, Ltd., Osaka, Japan), 8-week-old males (22–25 g) were injected intradermally with saline (one group) or 5 μg (10 μL) of mite allergen extract [*Dermatophagoides pteronyssinus* (Dp); Cosmo Bio LSL, Tokyo, Japan] dissolved in saline (five groups) on the ventral side of their right ears on treatment days 0, 2, 4, 7, 9, 11, 14, and 16 (Figure 1). Twenty-four hours after each intradermal injection, we measured ear thickness with a gauge (Ozaki Mfg. Co. Ltd., Osaka, Japan) and evaluated clinical scores for skin dryness, eruption, and wound, graded from 0 to 3 (0, no symptoms; 1, mild; 2, moderate; 3, severe). The untreated group received no Dp treatment.

### Histologic evaluation

Animals were sacrificed by etherization 24 hr after the last intradermal injection (day 17). Right ears of males were removed and fixed in 10% phosphate-buffered formalin (pH 7.2), embedded in paraffin, cut into 3-μm sections, and stained with hematoxylin/eosin (H&E) and toluidine blue (pH 4.0). We performed histologic analyses using an Olympus AX80 microscope (Olympus Corp., Tokyo, Japan) and measured the length of the cartilage in each specimen using an Olympus VM-30 video micrometer. The infiltration of eosinophils and mast cells were morphometrically evaluated as the number of cells per millimeter of cartilage. We also evaluated the degranulation of mast cells as not degranulated (0%), mildly degranulated (0–50%), and severely degranulated (> 50%) (Takano et al. 2006).

### ELISA

Right ears of males were removed 24 hr after the last intradermal injection; ears were then homogenized and centrifuged as previously described by Takano et al. (1997). We conducted enzyme-linked immunosorbent assays (ELISAs) for eotaxin (R&D Systems, Minneapolis, MN, USA) in the ear tissue supernatants according to the manufacturer’s instructions. The detection limit for eotaxin was < 3 pg/mL.

### Statistical analysis

We analyzed differences between the groups using Dunnett’s or Steel multiple comparison tests using Excel Statistics 2006 statistical software (Social Survey Research Information Co., Ltd., Tokyo, Japan). We considered *p*-values < 0.05 to be significant, and data are reported as mean ± SE.

## Results

### Maternal DEHP exposure during fetal periods and atopic dermatitis-like skin lesions in offspring

**Figure 2:**
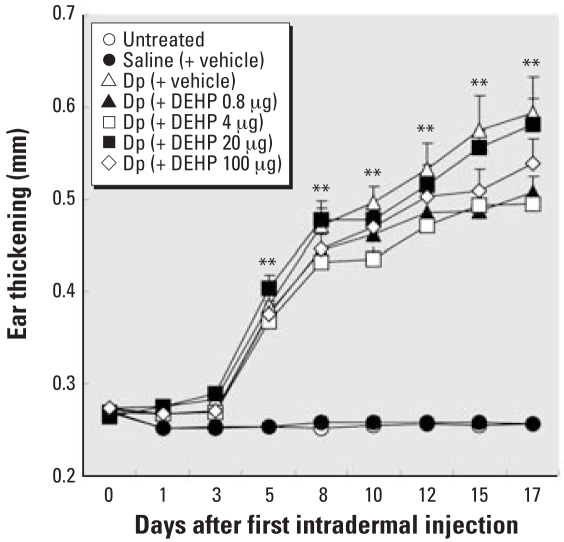
Effect of maternal exposure to DEHP during fetal periods on atopic dermatitis-like skin lesions in offspring shown by ear thickening 24 hr after each intradermal injection of Dp. Data shown are mean ± SE of 7–12 animals per group. **p < 0.01, Dp-treated groups compared with untreated group and saline (+ vehicle) groups.

To evaluate the effects of maternal exposure to DEHP during fetal periods on atopic dermatitis-like skin lesions related to mite allergen in males, we examined clinical scores, including dryness, eruption, wound, edema, and ear thickening in males in the presence or absence of mite allergen. Treatment with mite allergen significantly enhanced ear thickening (*p* < 0.01; Figure 2) and clinical scores (*p* < 0.01; see Supplemental Material, Figure 1, available online at http://www.ehponline.org/members/2008/11191/suppl.pdf) compared with untreated or saline groups from day 5. However, maternal exposure to DEHP during fetal periods did not show significant enhancing effects in the presence of allergen compared with exposure to vehicle alone. We found no change in the no treatment and saline treatment groups.

### Maternal DEHP exposure during neonatal periods and atopic dermatitis-like skin lesions in offspring

**Figure 3:**
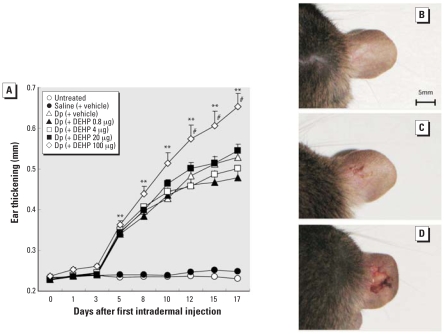
Effect of maternal exposure to DEHP during neonatal periods on atopic dermatitis-like skin lesions in offspring shown by ear thickening 24 hr after each intradermal injection of Dp (*A*) and macroscopic features 24 hr after the last injection (*B–D*). (*B*) Saline (+ vehicle). (*C*) Dp (+ vehicle). (*D*) Dp (+ DEHP 100 μg). Data shown are mean ± SE of 12 animals per group. **p < 0.01, Dp-treated groups compared with untreated and saline (+ vehicle) groups. ^#^*p* < 0.01, Dp (+ DEHP 100 μg) group compared with Dp (+ vehicle) group. For B–D, bar = 5 mm.

To evaluate the effects of maternal exposure to DEHP during neonatal periods on atopic dermatitis-like skin lesions related to mite allergen in males, we examined ear thickening and clinical scores. Treatment with mite allergen significantly enhanced ear thickening (*p* < 0.01; Figure 3A) and clinical scores (*p* < 0.01; see Supplemental Material, Figure 2, available online at http://www.ehponline.org/members/2008/11191/suppl.pdf) compared with untreated or saline groups from day 5. From day 12, maternal exposure to 100 μg DEHP significantly increased ear thickening (Figure 3A) and clinical scores (see Supplemental Material, Figure 2, available online at http://www.ehponline.org/members/2008/11191/suppl.pdf) in males compared with vehicle exposure in the presence of mite allergen. In macroscopic examination, we observed edema, dryness, excoriation, and crust at the dorsal site of allergen-injected ears in males (Figure 3C). Furthermore, these findings were prominent and were often accompanied by severe hemorrhage and erosion in the presence of maternal exposure to DEHP during neonatal periods (Figure 3D). We found no change in untreated (data not shown) or saline groups (Figure 3B).

### Maternal DEHP exposure during neonatal periods and histologic changes in the skin related to mite allergen in offspring

**Figure 4:**
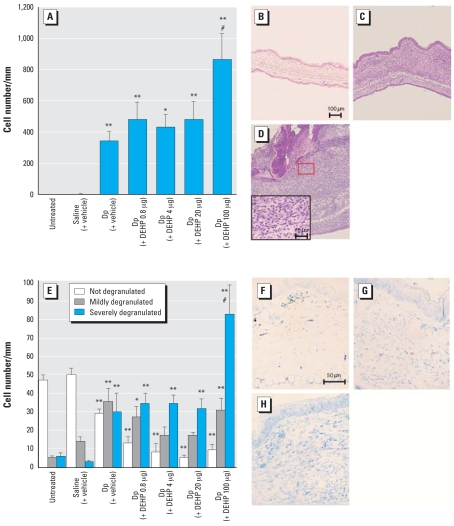
Histologic changes in the ear 24 hr after the last intradermal injection of Dp. The infiltration of eosinophils (*A*) and mast cells (*E*) were morphometrically evaluated as the number of cells per millimeter of cartilage. We also evaluated the degranulation of mast cells as not degranulated (0%), mildly degranulated (0–50%), and severely degranulated (> 50%). (*B–D*) and (*F–H*) show histologic findings of the saline (+ vehicle) (*B*, *F*), Dp (+ vehicle) (*C*, *G*), and Dp (+ DEHP 100 μg) (*D*, *H*) groups; tissues were stained with H&E (*B–D*) or toluidine blue (*F–H*). In *A* and *E,* data shown are mean ± SE of four animals per group. *p < 0.05, Dp-treated groups compared with untreated and saline (+ vehicle) groups. **p < 0.01, Dp-treated groups compared with untreated and saline (+ vehicle) groups. ^#^*p* < 0.01, Dp (+ DEHP 100 μg) group compared with Dp (+ vehicle) group. For *B–D*, bar = 100 μg; for inset in *D*, bar = 10 μm; for *F–H*, bar = 50 μm.

To determine the effects of maternal exposure to DEHP during neonatal periods on histologic changes of the skin related to mite allergen in males, we evaluated the skin specimens stained with H&E (Figure 4A–D) or toluidine blue (Figure 4E–H) 24 hr after the last intradermal inoculation. We found no pathologic alterations with no treatment or saline treatment (Figure 4B,4F). Treatment with mite allergen (Figure 4A,C) enhanced the infiltration of eosinophils into the skin lesions compared with saline treatment or no treatment (*p* < 0.01). Further, maternal exposure to 100 μg DEHP (Figure 4D) during neonatal periods caused more prominent eosinophilic inflammation than did exposure to vehicle in the presence of allergen [Figure 4A; *p* < 0.01 for Dp (+ DEHP 100 μg) group vs. Dp (+ vehicle) group]. In overall trend, these changes were consistent with the severity of mast cell degranulation [Figure 4E–H, *p* < 0.01 for Dp (+ DEHP 100 μg) group vs. untreated group, saline (+ vehicle) group, and Dp (+ vehicle) group].

### Maternal DEHP exposure during neonatal periods and protein expression of eotaxin in the skin related to mite allergen in offspring

**Figure 5:**
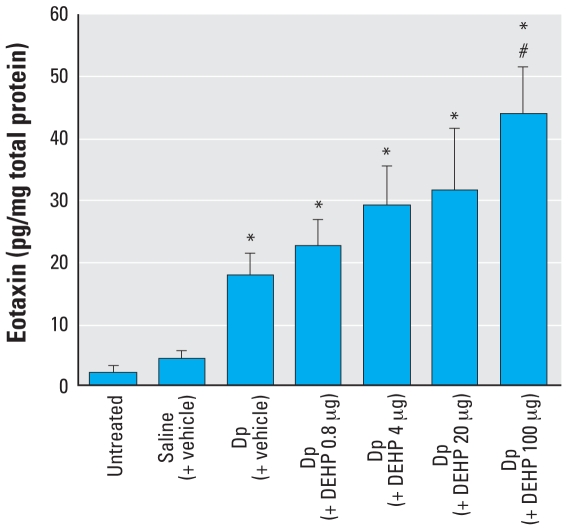
Effects of DEHP on local expression of eotaxin measured by ELISA 24 hr after the last intradermal injection of Dp. Data shown are mean ± SE of eight animals per group. *p < 0.05, Dp-treated groups compared with untreated and saline (+ vehicle) groups. ^#^*p* < 0.05, Dp (+ DEHP 100 μg) group compared with Dp (+vehicle) group.

We evaluated the protein expression of eotaxin in the ear 24 hr after the final intradermal inoculation to elucidate the mechanism of the enhancing effects of maternal exposure to DEHP during neonatal periods on atopic dermatitis-like skin lesions related to mite allergen in males. Treatment with mite allergen increased the expression of eotaxin compared with untreated or saline groups (Figure 5; *p* < 0.05). Furthermore, in males treated with mite allergen, maternal exposure to 100 μg DEHP during neonatal periods markedly enhanced the protein expression of eotaxin compared with vehicle exposure (*p* < 0.05). In addition, DEHP at a dose of 4 or 20 μg tended to enhance the local expression of eotaxin.

## Discussion

The present study shows that maternal exposure of mice to DEHP during neonatal periods aggravates atopic dermatitis-like skin lesions related to mite allergen in male offspring. The enhancing effects are nearly paralleled by those on eosinophilic inflammation, mast cell degranulation, and local expression of eotaxin. In contrast, we observed no significant enhancing effects after maternal exposure to DEHP during fetal periods.

DEHP, the most commonly used plasticizer in flexible PVC formulations, is a ubiquitous environmental toxicant. Thus, the general population can be exposed to DEHP in food, water, and air via ingestion or inhalation. In particular, fetuses and infants can be maternally exposed to DEHP at early life stages (Calafat et al. 2004; Latini et al. 2003a, 2003b; Main et al. 2006). Previous studies have suggested that maternal exposure to phthalates during fetal and/or neonatal periods can adversely affect reproduction and development by acting as EDCs in animals and humans (Borch et al. 2006; Kim et al. 2004; Marsee et al. 2006). However, whether maternal exposure to DEHP and/or its metabolites during fetal and/or neonatal periods can affect allergic diseases, including atopic dermatitis in offspring, has not been elucidated.

The developing immune system, which depends chiefly on mother-derived humoral immunity during embryonic/fetal periods and acquired cellular and humoral immunity during neonatal periods, can be vulnerable to environmental toxicants such as EDCs (Holladay 1999; Holladay and Smialowicz 2000). Epidemiologic studies have indicated that exposure to EDCs, such as dioxins and polychlorinated biphenyls, during human development can affect the immune system (Dallaire et al. 2004; Nagayama et al. 1998; Weisglas-Kuperus et al. 1995). In experimental studies, prenatal/perinatal exposure to 2,3,7,8-tetrachlorodibenzo-*p*-dioxin and bisphenol A can modulate immune responses in rodents (Gehrs et al. 1997; Vorderstrasse et al. 2006; Yoshino et al. 2004). On the other hand, few studies have exposed experimental animals to environmental chemicals only during postnatal periods. In the present study, maternal exposure to DEHP only during neonatal periods enhanced atopic dermatitis-like skin lesions related to mite allergen in male offspring. In contrast, maternal exposure to DEHP during fetal periods did not affect the aggravation of atopic dermatitis-like skin lesions related to mite allergen. Together, these data indicate that maternal exposure to DEHP may contribute to the modulation of adaptive immunity in offspring rather than mother-derived immunity in our model. This hypothesis is supported by our previous study in which DEHP exposure in young subjects enhanced atopic dermatitis-like skin lesions related to mite allergen (Takano et al. 2006). Our present and previous results thus implicate exposure to environmental chemicals during neonatal periods in the enhancement of allergen-related inflammation in offspring.

DEHP reportedly induces reproductive and developmental problems predominantly in male rodents (Borch et al. 2006; Kim et al. 2004). Also, a recent epidemiologic study has suggested that estimated daily phthalate exposure is associated with reduced anogenital distance in male infants (Marsee et al. 2006). Our present experiments also showed that maternal exposure to DEHP during fetal and neonatal periods did not affect atopic dermatitis-like symptoms in females (data not shown). The present results may demonstrate the existence of sex differences in the sensitivity to DEHP during neonatal periods in the case of atopic dermatitis, similar to differences in aromatase activity described by Andrade et al. (2006).

In the present study, maternal exposure to DEHP during neonatal periods aggravates atopic dermatitis-like skin lesions in male offspring at a dose about 30-fold lower (100 μg/ animal/week ≈ 600 μg/kg/day) than the no observed adverse effect level (19 mg/kg/day), which was determined on the basis of histologic changes in the rodent liver (Carpenter et al. 1953). Phthalate metabolites have been found in human breast milk (Calafat et al. 2004; Main et al. 2006) and in infant formula (Mortensen et al. 2005; Sorensen 2006). Several studies have estimated the average daily intake of DEHP in infants 0–3 months of age at 13 μg/kg/day for infant formula and 21 μg/kg/day for breast milk (Latini et al. 2004). On the other hand, there are differences in disposition kinetics of DEHP and its biological metabolite MEHP between intraperitoneal and oral administration (Pollack et al. 1985). In addition, oral exposure of rat dams to DEHP at high doses (2 g/kg for 5 days) during lactation can lead to plasma levels of DEHP (216 ± 23 μg/mL, mean ± SE) and MEHP (25 ± 6 μg/mL) in suckling pups (Dostal et al. 1987). To our knowledge, however, no previous studies have determined the differences in the plasma levels of suckling pups and/or breast milk levels of DEHP and its metabolites after maternal exposure to DEHP via different routes during neonatal periods. Thus, in the next stage of research, we should try to quantify the concentration of DEHP and/or its metabolites in the breast milk and/or plasma levels of suckling pups after intraperitoneal or oral administration and investigate the effects of oral administration at the level of human exposure of DEHP.

In the present study we found that maternal exposure to DEHP at a dose of 100 μg during neonatal periods increased the expression of eotaxin in the ear tissue in the presence of allergen. The results were concomitant with the recruitment of eosinophils into skin lesions and with mast cell degranulation. Eosinophils play a prominent proinflammatory role in a broad range of allergic diseases, including atopic dermatitis. Eotaxin is important in the early recruitment of eosinophils after allergen challenge. Eotaxin is reportedly increased in the blood of patients with atopic dermatitis (Hossny et al. 2001; Jahnz-Rozyk et al. 2005) and expressed in lesions of human atopic dermatitis (Yawalkar et al. 1999). CCR3, a principal receptor for eotaxin, has been found to be essential for eosinophil recruitment into murine skin lesions caused by repeated allergen sensitization (Ma et al. 2002). In addition, tryptase/chymase double-positive mast cells express CCR3 and are attracted by eotaxin (Ochi et al. 1999; Romagnani et al. 1999). Also, mast cell–deficient mice have shown reduced lung eosinophilia after eotaxin administration compared with wild-type mice after allergen sensitization (Das et al. 2006). Interestingly, the present study shows that DEHP at a dose of 4 or 20 μg tended to enhance the local expression of eotaxin. These findings indicate that maternal exposure to DEHP at doses < 100 μg during neonatal periods can prompt the manifestation of atopic dermatitis in offspring at the molecular level. In contrast, mite allergen treatment induced the expression of T helper 2 (T_H_2)-type cytokines, including IL-4, IL-5, IL-13, and RANTES, compared with no treatment or saline treatment, whereas maternal exposure to DEHP during neonatal periods did not significantly enhance the effects (data not shown). Thus, eotaxin might be a critical molecule in our model.

In our present model using NC/Nga mice, mite allergen treatment significantly elevated the production of total IgE and allergen-specific IgG1 in serum, increased the number of submandibular lymph node cells, and enhanced cell proliferation of submandibular lymph node cells in the presence of allergen stimulation *ex vivo* (data not shown). These results suggest that allergen-specific responses play critical roles in the present model of atopic dermatitis-like skin lesions. In addition, maternal exposure to DEHP during neonatal periods enhanced effects related to mite allergen in offspring, including eotaxin expression, eosinophilic inflammation, and mast cell degranulation, all of which are typically shown in allergen-related T_H_2-dominant responses and/or inflammation (Heishi et al. 2003; Sasakawa et al. 2001; Yagi et al. 2002; Yamashita et al. 2007). Taken together, maternal exposure to DEHP during neonatal periods can accelerate allergen-related inflammation possibly via T_H_2-dominant responses in our model.

## Conclusion

In the present study using a murine model, we have shown that maternal exposure to DEHP during neonatal periods aggravates atopic dermatitis-like skin lesions related to mite allergen in male offspring. The enhancing effects may be mediated through the enhanced expression of eotaxin. Our results support the novel hypothesis that maternal exposure to DEHP during neonatal periods via breast milk and/or infant formula may be responsible, at least in part, for the recent increase in atopic dermatitis in offspring. To further clarify this hypothesis, we should examine breast milk levels and/or suckling pup plasma levels of DEHP and/or its metabolites and the time course of cytokine expression in our model, as well as in a model using oral administration of DEHP. Furthermore, epidemiologic studies should determine the relationships between exposure to DEHP via breast milk and/or infant formula and the manifestation of atopic dermatitis in offspring.
